# Different Responses to Drug Safety Screening Targets between Human Neonatal and Infantile Heart Tissue and Cardiac Bodies Derived from Human-Induced Pluripotent Stem Cells

**DOI:** 10.1155/2019/6096294

**Published:** 2019-03-06

**Authors:** Jan Trieschmann, Moritz Haustein, Annette Köster, Jürgen Hescheler, Konrad Brockmeier, Gerardus Bennink, Tobias Hannes

**Affiliations:** ^1^Department of Paediatric Cardiology, Heart Centre Cologne, University Hospital of Cologne, Kerpener Str. 52, 50924 Cologne, Germany; ^2^Institute for Neurophysiology, University of Cologne, Robert-Koch-Str. 39, 50931 Cologne, Germany; ^3^Cardiothoracic Surgery, University Hospital of Cologne, Kerpener Str. 52, 50924 Cologne, Germany; ^4^Department of Paediatric Critical Care Medicine and Neonatology, University Hospital of Cologne, Kerpener Str. 52, 50924 Cologne, Germany

## Abstract

**Aims:**

Induced pluripotent stem cell-derived cardiomyocytes (iPS-CMs) have become a promising tool in cardiovascular safety pharmacology. Immaturity of iPS-CMs remains an ongoing concern. We compared electrophysiological and contractile features of cardiac bodies (hiPS-CBs) derived from human-induced pluripotent stem cells and human neonatal and infantile myocardial slices relevant for drug screening.

**Methods and Results:**

Myocardial tissue slices were prepared from biopsies obtained from patients undergoing surgery for hypoplastic left heart syndrome (HLHS) and tetralogy of Fallot (TOF). Electrophysiological features and response to I_k,r_ blockade as well as contractile properties were investigated using microelectrodes and isometric force measurements and were compared to hiPS-CBs. Both native myocardial tissue slices as well as hiPS-CBs showed action potential prolongation after I_k,r_ blockade, but early afterdepolarisations could be observed in native myocardial tissue slices only. The force-frequency relationship (FFR) varied at lower frequencies and was negative throughout at higher frequencies in hiPS-CBs. In contrast, native myocardial tissue slices exhibited positive, negative, and biphasic FFRs. In contrast to native myocardial tissue slices, hiPS-CBs failed to show an inotropic response to *ß*-adrenergic stimulation. Although all groups showed *ß*-adrenergic induced positive lusitropy, the effect was more pronounced in myocardial tissue slices.

**Conclusion:**

hiPS-CBs were able to reproduce AP prolongation after I_k,r_ blockade, but to a lesser extent compared to human neonatal and infantile myocardial tissue slices. Early afterdepolarisations could not be induced in hiPS-CBs. Contractile force was differently regulated by *β*-adrenergic stimulation in hiPS-CBs and the native myocardium. If used for cardiotoxicity screening, caution is warranted as hiPS-CBs might be less sensitive to pharmacologic targets compared to the native myocardium of neonates and infants.

## 1. Introduction

Developing assays to predict cardiotoxicity has become one of the most important goals in drug discovery and development in order to anticipate potential cardiovascular side effects. Failure of animal models to detect drug-induced cardiotoxicity has led to recognition of cardiovascular side effects during the late phase of development or after introduction into the market. This did not only put patients at risk but also led to costly drug recalls [1]. Improving the predictive power of cardiotoxicity assays has therefore become one of the priorities of pharmacological research in recent years. To avoid the fallacies of animal models in the past, careful gauging of cardiotoxicity assays to human cardiomyocyte physiology is required.

Failure of animal models to recognise arrhythmogenicity of drugs can be explained by the notorious species dependency of cardiac repolarisation. Inhibition of the rapid component of the inward rectifying potassium current I_k,r_ plays a central role in human cardiac repolarisation and has been established as an important cause of drug-related ventricular arrhythmia and sudden cardiac death [2]. The relevance of I_k,r_ inhibition is greatly underappreciated in rodent models of cardiac electrophysiology [3]. Even well-established models of cardiac electrophysiology such as the canine ventricular myocardium show important differences to their human counterpart in ionic mechanisms underlying repolarisation [4].

Use of cardiomyocytes derived from human pluripotent stem cells for drug safety screening seems to be an obvious solution to this problem. Somatic cells can be reprogrammed to induced pluripotent stem cells (iPSCs) [5], and these can be differentiated to induced pluripotent stem cell-derived cardiomyocytes (iPS-CMs) [6, 7]. iPS-CMs can be generated from patients with inherited channelopathies [8]; thus, cardiotoxicity screening might be performed specifically for high-risk patients. However, an obstacle to using iPS-CMs for cardiotoxicity screening is their immature electrophysiological and contractile behaviour [9].

Adding to the problem of species-dependent differences of cardiac electrophysiology, repolarisation underlies a significant variability within a single species that is further accentuated by developmental changes and disease. To give an example, in a recent study the repolarising currents I_k,s_ and I_t,o_ were shown to be present in the adult canine heart but absent in the neonatal canine left ventricle [10].

Thus, when designing cardiotoxicity assays, one needs to take age-specific changes of cardiac function into account. A major obstacle to designing cardiotoxicity assays for the paediatric population is simply the paucity of data available for cardiac cellular physiology in the young.

Therefore, in the present study, we aimed to compare (1) action potential configuration, (2) response to I_k,r_ blockade, (3) intrinsic regulation of contractile force by frequency, and (4) extrinsic regulation of force by *β*-adrenergic stimulation between neonatal and infantile heart tissue and iPS-CM tissue constructs after prolonged culture.

## 2. Methods

### 2.1. Patients

The ventricular myocardium of patients undergoing heart surgery was only collected when removal of tissue was required for surgical reasons. This was (1) for insertion of a right ventricle to pulmonary artery shunt in the first stage of palliation of hypoplastic left heart syndrome (HLHS) [Sano S 2003] and (2) for relieve of right ventricular outflow tract (RVOT) obstruction during corrective surgery for tetralogy of Fallot (TOF).

Collection of tissue samples complied with the World Medical Association Declaration of Helsinki (7th Revision, Fortaleza, Brazil, 2013) and was approved by the local ethics committee at the University of Cologne (reference no. 07-045). Informed consent was obtained from the caregivers of every patient.

### 2.2. Preparation of Myocardial Tissue Slices

Immediately after excision by the surgeon, ventricular myocardial tissue was immersed into ice-cooled, oxygenated low Ca^2+^ Tyrode's solution (composition in mmol/L: NaCl 136, KCl 5.4, NaH_2_PO_4_ 0.33, MgCl_2_ 1, glucose 10, HEPES 5, CaCl_2_ 0.05, BDM 30; insulin 20 mU; pH 7.4 adjusted with NaOH). Samples were transported to the laboratory (transport time: 18.2 ± 3.6 minutes, *n* = 16, mean ± SD) under continuous oxygenation and cooling. On arrival in the laboratory, tissue chunks were further dissected into smaller blocks before slicing.

For preparation of myocardial slices, ventricular tissue blocks were embedded with liquescent 4% low-melting agarose maintained at 37°C (Carl Roth, Karlsruhe, Germany). After solidification of the agarose on ice, tissue blocks were sectioned with steel blades (Campden Instruments, Leicester, England) into 300 *μ*m thick tissue slices using a vibratome (VT1000 S; Leica Microsystems, Wetzlar, Germany).

After collecting the tissue slices, Ca^2+^ tolerance was induced by raising the extracellular Ca^2+^ concentration over 30 min to 0.95 mmol/L in two steps. Subsequently, slices were transferred into cold (4°C) Iscove's modified Dulbecco's medium (IMDM; Life Technologies, Darmstadt, Germany) supplemented with 20% foetal calf serum (FCS; Life Technologies). Temperature was raised slowly to 37°C in an incubator for at least 1 h (Supplemental Figure 1).

### 2.3. Human-Induced Pluripotent Stem Cell-Derived Cardiomyocyte (hiPS-CM) Bodies

hiPS-CM cardiac bodies (hiPS-CBs) were purchased from Axiogenesis (Cologne, Germany, http://www.axiogenesis.com). Custom-made cardiac bodies were produced from CorV.4U ventricular enriched cardiomyocytes and FibroCor.4U cardiac fibroblasts (cFBs) (Supplemental Figure 1). For electrophysiological recordings, cardiac bodies were formed using the hanging drop method (30 k hiPS-CMs and 13 k cFBs). As these were not suitable for force measurements, we used a second differentiation protocol to generate hiPS-CBs for force measurements. A specialised extracellular matrix- (ECM-) based production was applied to obtain 3D constructs large enough for force measurements (250 k hiPS-CMs and 100 k cFBs). Cardiac bodies were cultured with Cor.4U culture medium supplemented with ciprofloxacin (Axiogenesis). The medium was changed every 3 to 4 days. hiPS-CBs were used for experiments after 9 weeks or 16 weeks of differentiation in accordance with studies reporting an improved maturation status of hiPS-CMs after prolonged culture [9, 11].

### 2.4. Action Potential Recordings

Tissue slices or hiPS-CBs were superfused with Dulbecco's modified Eagle's medium (DMEM; Life Technologies). Temperature was kept at 37°C, and oxygen tension and pH were maintained by constant bubbling of the superfusate with carbogen (95% O_2_; 5% CO_2_). For intracellular recordings, conventional glass microelectrodes (WPI, Sarasota, USA) with resistances between 20 and 50 MΩ were filled with 3 mol/L KCl. Action potentials were recorded by a SEC-10LX amplifier (NPI Electronic, Tamm, Germany) using the Pulse software (Heka, Lambrecht/Pfalz, Germany). The beating frequency was controlled using an SD9 square pulse stimulator (Grass Technologies, West Warwick, USA) with a unipolar stimulation electrode. After steady-state conditions were achieved, drugs were administered for at least 10 minutes. The action potential duration to 20%, 50%, or 90% of repolarisation (APD20, APD50, and APD90), amplitude, maximal diastolic potential (MDP), and maximal upstroke velocity (*V*_max_) were analysed offline with Mini Analysis software (Synaptosoft, Fort Lee, USA).

### 2.5. Isometric Force Measurements

Slices and hiPS-CBs were mounted onto J-shaped steel needles connected to an isometric force transducer (Scientific Instruments, Heidelberg, Germany) and afterwards immersed in a chamber filled with IMDM without serum (1.97 mmol/L Ca^2+^). Temperature was maintained at 37°C, and the solution was continuously bubbled with carbogen (95% O_2_; 5% CO_2_). Field stimulation was performed by silver electrodes connected to a custom-made stimulator (10.7-23.5 V, pulse duration 5 ms). The length of specimens was increased stepwise to the length of maximal force development during continuous stimulation at 2 Hz. Analogue signals from the force transducer (KG7A; range 0–5 mN, resolution 0.2 N, resonance frequency 250–300 Hz) were amplified with a bridge amplifier (BAM7C; Scientific Instruments). Signals were digitised at a sampling frequency of 1 kHz. Recording and offline analysis were performed by using DASYLab software version 7.0 (National Instruments, Munich, Germany).

To determine the force-frequency relationship (FFR), specimens were stimulated following the protocol of alternating higher and lower frequencies (2.0, 1.5, 2.5, 1.0, 3.0, 0.5, and 4.0 Hz for 2 minutes each). Isoproterenol experiments were performed during constant field stimulation at 2 Hz except for 16 wk hiPS-CBs which were paced at 3 Hz due to their higher intrinsic automaticity. Baseline conditions and subsequent dose applications were recorded for 10 minutes each. For data analysis, signals were filtered (high- and low-pass Bessel filters: 0.05 Hz, 15 Hz, 8th order) and baseline correction was achieved by subtraction of a moving arithmetic average of 10,000 samples. To analyse the amplitude, time to peak, and time to 50% and 90% relaxation, single contractions were averaged during steady-state conditions (30 to 300 seconds before the next experimental step) using an automated algorithm.

### 2.6. Solutions

All chemicals for buffer and stock solution preparation were purchased from Sigma-Aldrich (Steinheim, Germany), unless otherwise stated. The E4031 (Abcam, Cambridge, UK) stock was prepared in Aqua Dest at a concentration of 1 mM. (-)-Isoproterenol (ISO) was dissolved in Aqua Dest at a concentration of 10 mM with 10 mM ascorbic acid.

### 2.7. Statistics

For statistical analysis and data visualisation, SPSS version 24 (IBM, Armonk, USA) and GraphPad Prism version 4 (GraphPad Software, San Diego, USA) were used. Results are represented as mean with 95% confidence intervals (CIs), if not otherwise stated. CIs for EAD occurrence were calculated using the recommended method [12] as it performs well for small sample sizes and when no events are observed. Normality of data sets was tested using the Shapiro-Wilk procedure. The effect of treatments in an individual group was statistically analysed by one-way ANOVA with repeated measures or Friedman's ANOVA for normal and nonnormal distributed data sets, respectively. For repeated measures ANOVA procedure, Greenhouse-Geisser correction was applied when assumption of sphericity was violated (Mauchly's test), and Tukey LSD post hoc testing was carried out for pairwise comparisons. Wilcoxon rank tests were used for multiple comparisons after significant Friedman's ANOVA results. Differences between groups were statistically analysed using one-way ANOVA or Kruskal-Wallis procedure as appropriate. To identify which groups differ statistically, Hochberg (equal variances) or Games-Howell (unequal variances) post hoc tests were performed after significant ANOVAs. Variance homogeneity was checked with the Levene procedure. Mann-Whitney *U* pairwise testing was performed after significant Kruskal-Wallis results. Paired *t*-tests were applied when only two experimental conditions were compared.

All significance tests were two-tailed; statistical significance was assumed for *p* < 0.05.

## 3. Results

### 3.1. Patients

In total, 17 patients were included. Of the 7 patients undergoing the Norwood procedure with implantation of a RV-PA shunt, 6 had a diagnosis of HLHS; the remaining patient had interrupted aortic arch with ventricular septal defect.

The TOF group consisted of 10 patients of whom eight underwent TOF repair with closure of ventricular septal defect and patch annuloplasty of the pulmonary artery. Of these eight patients, one had a previous RVOT stent and three patients had additional ligation of persistent ductus arteriosus. The two remaining patients underwent implantation of a valved RV-PA conduit, closure of ventricular septal defect, and RVOT myectomy for a diagnosis of TOF with pulmonary atresia and after aortopulmonary shunt. Patient characteristics are listed in Table 1.

### 3.2. AP Configuration

**Figure 1:**
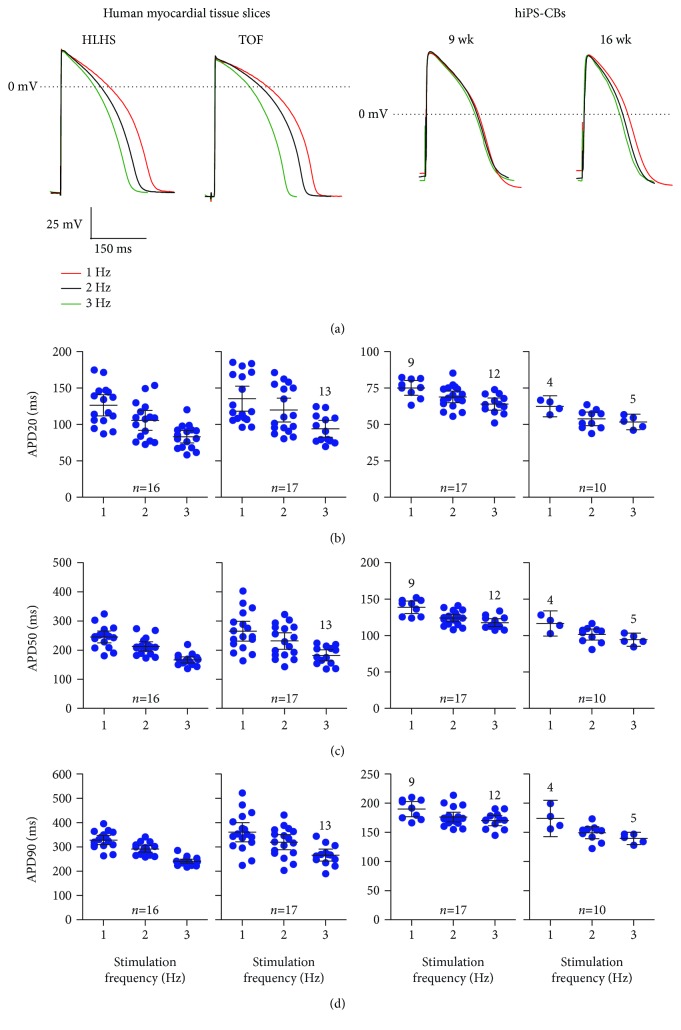
Frequency adaptation of APD. (a) Original traces of microelectrode recordings in myocardial tissue slices of patient HLHS4 and TOF1 and hiPS-CBs (9 and 16 wks). (b-d) APD at different stimulation frequencies at 20% (b), 50% (c), and 90% (d) repolarisation. Data are expressed as mean ± 95% CI. Numbers in the graphs indicate deviations from reported overall *n* numbers at individual experimental steps. The *y*-axis differs between myocardial tissue slices and hiPS-CBs.

Compared at a stimulation frequency of 2 Hz, TOF showed longer APD50 and APD90 as well as lower *V*_max_ than did HLHS. In hiPS-CBs, APD20, APD50, and APD90 were shorter compared to tissue slices (Figure 1, Supplemental Table 1).

Both TOF and HLHS preparations showed a rate-dependentadaptation of action potential duration at 20%, 50%, and 90% repolarisation (Figure 1, Supplemental Table 1). All HLHS preparations could be stimulated up to 3 Hz and showed frequency-dependent shortening of action potential duration. Four of 17 TOF preparations failed to respond at a stimulation rate of 3 Hz.

hiPS-CBs showed an intact rate-dependent adaptation of action potential duration (Figure 1, Supplemental Table 1). APD20, APD50, and APD90 were shorter in both 6 wk and 16 wk hiPS-CBs compared to tissue slices (Figure 1, Supplemental Table 1).

None of the preparations showed afterdepolarisations under baseline conditions.

### 3.3. AP Response to I_k,r_ Blockade by E4031

**Figure 2:**
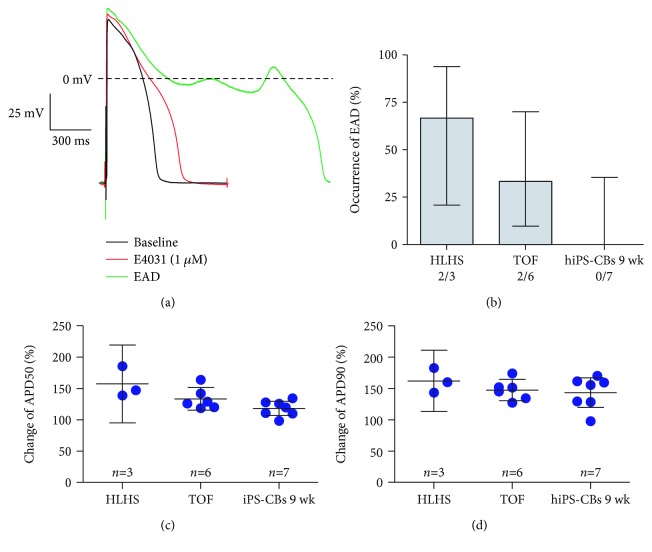
Effects of E4031 1 *μ*M on AP duration and occurrence of EADs. (a) Representative traces of patient TOF3 before (black) and during superfusion with E4031 1 *μ*M before (red) and after (green) occurrence of EADs. (b) Occurrence of EADs in HLHS tissue slices, TOF tissue slices, and hiPS-CBs (9 wk) during superfusion with E4031 1 *μ*M. (c-d) Change of APD50 (c) and APD90 (d) during administration of E4031 1 *μ*M relative to baseline. Data are expressed as mean ± 95% CI. Numbers above error bars indicate *p* values <0.05 vs. HLHS (1), TOF (2), and 9 wks hiPS-CBs (3). Effect on APD50: ANOVA *p* = 0.016; Hochberg post hoc: HLHS vs. TOF: *p* = 0.17; HLHS vs. hiPS-CBs 9 wk: *p* = 0.014; TOF vs. hiPS-CBs: *p* = 0.32; and effect on APD90: ANOVA *p* = 0.47.

TOF (*n* = 6) and HLHS (*n* = 3) tissue preparations as well as 6 wk hiPS-CBs (*n* = 7) showed a considerable increase of APD50 and APD90 after application of E4031 1 *μ*M before the occurrence of EADs (Figures 2(a), 2(c), and 2(d)). The effect was most pronounced in HLHS myocardial slices increasing APD50 by 156% and APD90 by 162%. In TOF preparations, APD50 increased by 131% and APD90 by 142%. EADs occurred in 2/3 HLHS preparations and 2/6 TOF preparations (Figure 2(b)). Consistent with native myocardial tissue, APD50 increased in hiPS-CBs by 117% and APD90 by 141%. In contrast to native myocardial tissue, afterdepolarisations were not observed in iPS-CBs (0/7) (Figure 2(b)).

**Figure 3:**
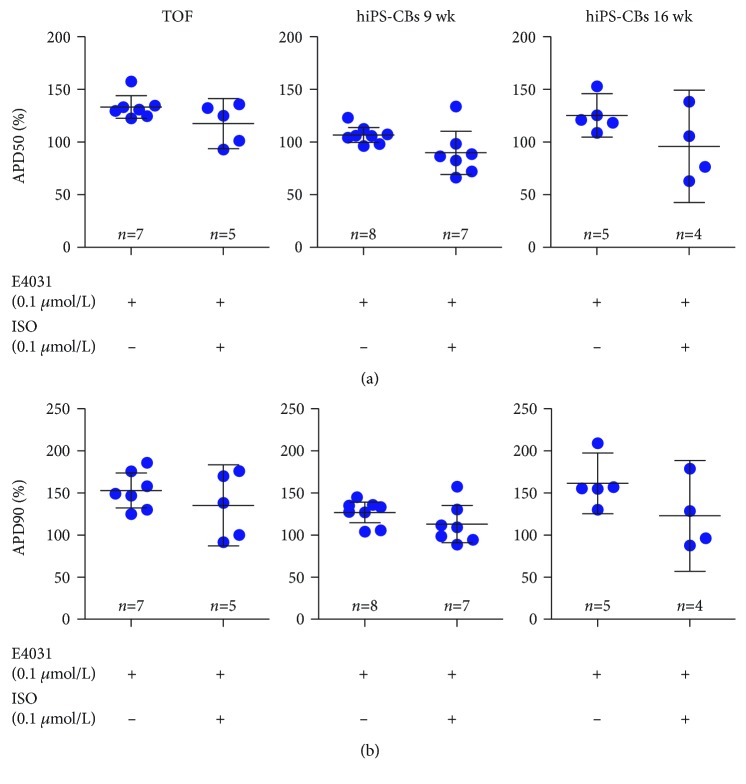
Effects of E4031 0.1 *μ*M and ISO 0.1 *μ*M on APD50 (a) and APD90 (b) in TOF tissue slices, iPS-CBs (9 wk), and iPS-CBs (16 wk). Data are expressed as mean ± 95% CI.

To test whether *ß*-adrenergic stimulation facilitates afterdepolarisations after I_k,r_ blockade, we sequentially superfused TOF tissue slices and hiPS-CBs with E4031 0.1 *μ*M in combination with ISO 0.1 *μ*M. The application of E4031 0.1 *μ*M led to a prolongation of APD50 and APD90 in both TOF tissue slices by 134% and 152%, respectively. In hiPS-CBs, APD90 increased slightly by 126%, while APD50 remained unchanged (Figure 3). We did not observe early afterdepolarisations at this lower concentration.

After superfusion with both E4031 0.1 *μ*M and ISO 0.1 *μ*M, we observed a shortening of APD in both TOF tissue slices and cardiac bodies. In 3/7 TOF tissue slices, EADs occurred after superfusion with both drugs. We did not observe afterdepolarisations in hiPS-CBs.

Detailed statistics for all experiments are presented in Supplemental Table 2.

### 3.4. Contractile Force and Force-Frequency Relationship (FFR)

**Figure 4:**
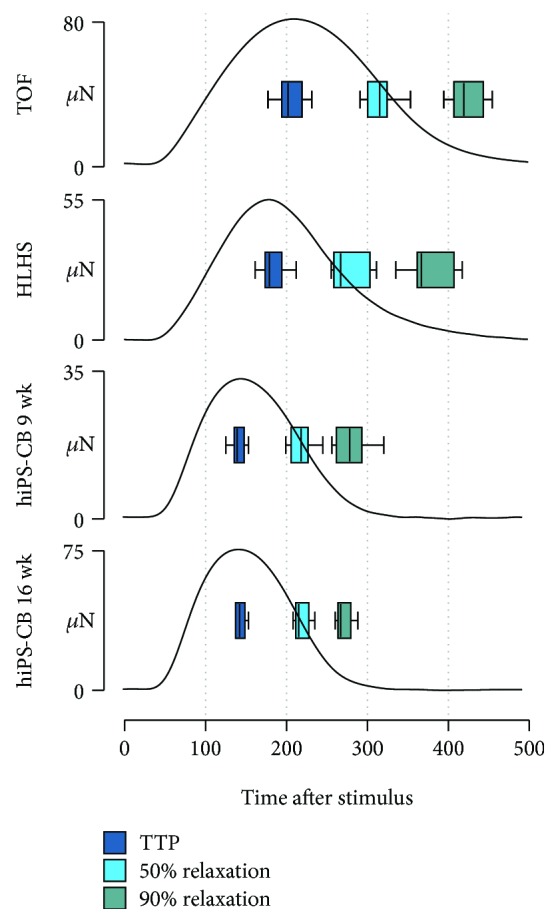
Duration of contractions. Representative averaged twitches from a myocardial slice of patients TOF8 and HLHS7 as well as hiPS-CBs (9 and 16 wk) at a stimulation frequency of 2 Hz. Boxplots display the distribution of time to peak (TTP) as well as time to 50% and 90% relaxation in the groups (TOF, *n* = 19 of 8 patients; HLHS, *n* = 10 of 6 patients; 9 wk hiPS-CBs, *n* = 12; and 16 wks hiPS-CBs, *n* = 5).

The duration of twitches was substantially longer in human myocardial tissue slices than in hiPS-CBs (Figure 4, Supplemental Table 3). Time to peak as well as time to 50% and 90% relaxation were longer in tissue slices for all tested frequencies except at 4 Hz. At a stimulation frequency of 2 Hz, TOF tissue slices exhibited the longest durations followed by HLHS tissue slices with slightly shorter durations. In contrast, 9 wk hiPS-CB and 16 wk hiPS-CB durations were similar to each other but shorter compared to tissue slices.

**Figure 5:**
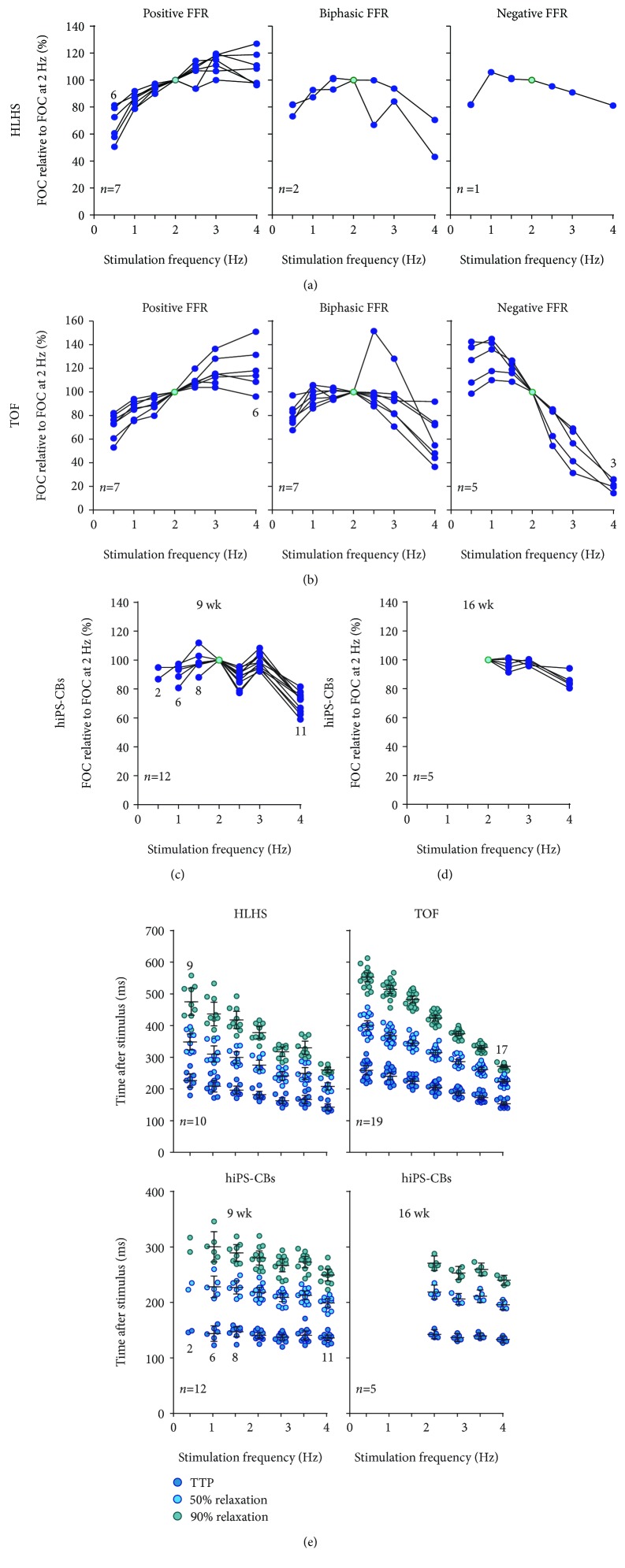
Force-frequency relationship (FFR). (a-d) Force of contraction (FOC) at different stimulation frequencies relative to FOC at 2 Hz for myocardial slices from HLHS (a) and TOF (b) patients as well as for 9 wk (c) and 16 wk (d) old hiPS-CBs. Single measurements from HLHS and TOF tissue slices were further subdivided in positive, biphasic, and negative FFRs (*y*-axis identical within the same group). However, such a separation of FFR pattern was not feasible for hiPS-CBs. (e) Frequency-dependent effects on time to peak (TTP) as well as time to 50% and 90% relaxation of myocardial tissue slices and hiPS-CBs. The *y*-axis differs between myocardial tissue slices and hiPS-CBs. Numbers in the graphs indicate deviations from reported overall *n* numbers at individual experimental steps (TOF, *n* = 19 of 8 patients; HLHS, *n* = 10 of 6 patients; 9 wk hiPS-CBs, *n* = 12; and 16 wk hiPS-CBs, *n* = 5).

For human myocardial tissue slices, FFRs could be subdivided in positive, negative, and biphasic patterns (Figures 5(a) and 5(b)). Positive FFRs showed a constant frequency-dependent increase of force of contraction between 0.5 Hz and 2 Hz which either continued to increase or did not decline at higher stimulation frequencies. In contrast, negative FFRs were featured by a frequency-dependent decline of force of contraction although the developed force was higher at 1 Hz compared to 0.5 Hz in almost all measurements. Biphasic FFRs showed an increasing trend of force of contraction at lower stimulation frequencies which was reversed at higher frequencies.

In HLHS myocardial tissues (Figure 5(a)), 7 slices of 3 patients showed a positive FFR, 2 slices of 2 patients exhibited a biphasic pattern, and a negative FFR could be only observed in 1 tissue slice of 1 patient. In contrast, single slices from the same TOF patient showed different FFR characteristics in some cases (Figure 5(b)). Slices from 2 patients showed only positive FFRs (*n* = 1 each), slices from 1 patient exhibited only negative FFRs (*n* = 2), and slices from 2 patients showed biphasic FFRs (*n* = 1 and 2). However, slices from 1 patient showed positive and biphasic FFRs (*n* = 4), slices from 1 patient revealed negative and biphasic FFRs (*n* = 4), and slices from 1 patient showed all three types of FFRs (*n* = 4).

Due to their intrinsic beating rate, contractile force could not be determined for all tested frequencies in all cardiac bodies. In particular, none of the examined 16 wk hiPS-CBs could be stimulated properly at frequencies lower than 2 Hz. Moreover, FFR behaviour of cardiac bodies did not conform to the previous described patterns observable in myocardial tissue slices. 9 wk hiPS-CBs showed an alternating trend of increase and decrease in force of contraction across ascending stimulation frequencies which was particularly pronounced at frequencies above 2 Hz (Figure 5(c)). Interestingly, stimulation at 4 Hz resulted in a strong decline in force of contraction compared to lower stimulation frequencies. In the range of successful electrical stimulation, 16 wk hiPS-CBs exhibited an almost flat FFR between 2 Hz and 3 Hz, but the force of contraction was slightly reduced in 4 of 5 cardiac bodies at 4 Hz (Figure 5(d)).

All groups demonstrated a frequency-dependent acceleration of relaxation (FDAR) (Figure 5(e)). However, FDAR was more pronounced in the myocardial tissue slices than in hiPS-CBs (Supplemental Table 3; Supplemental Figure 2). At a stimulation frequency of 4 Hz, time to 90% relaxation was reduced by ~36% and ~31% for TOF and HLHS tissue slices, respectively, compared to contractions at 2 Hz. In contrast, a reduction of only ~11% was found in hiPS-CBs (Supplemental Figure 3).

### 3.5. Effect of *ß*-Adrenergic Stimulation on Inotropy and Lusitropy

**Figure 6:**
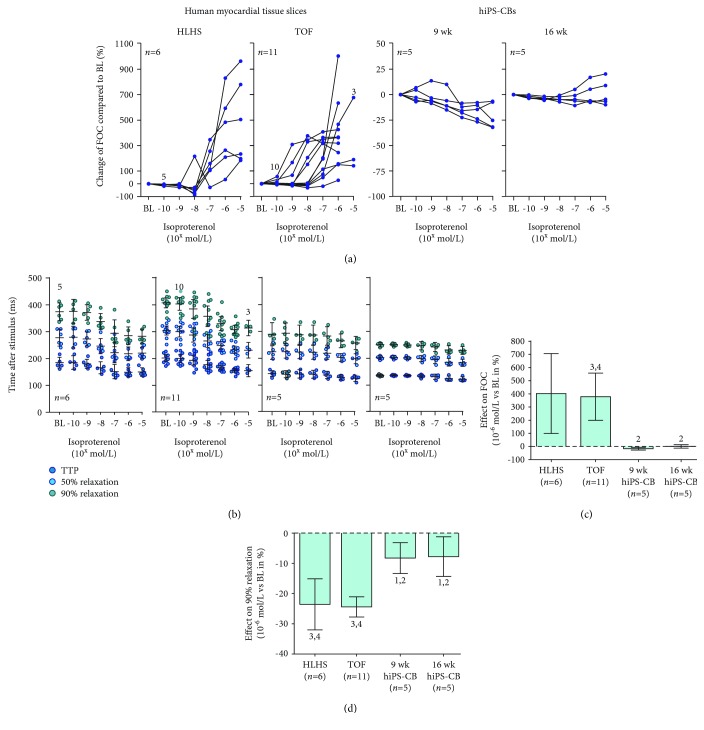
Response to *ß*-adrenergic stimulation by ISO. (a-b) Effect of ascending ISO concentrations in single experiments with human myocardial tissue slices (HLHS: *n* = 6 of 5 patients, left panels; TOF: *n* = 11 of 5 patients, middle left panels) and hiPS-CBs (9 wk: *n* = 5, middle right panels, 16 wk: *n* = 5, right panels). Numbers in the graphs indicate deviations from reported overall *n* numbers at individual experimental steps. (a) Change in force of contraction (FOC) in relation to baseline (BL). The *y*-axis differs between myocardial tissue slices and hiPS-CBs. (b) Effect on time to peak (TTP) as well as time to 50% and 90% relaxation. Lines and error bars represent the mean and 95% CIs, respectively. The *y*-axis is the same for all groups. (c-d) Comparison of average effect sizes of 10.6 mol/L ISO relative to baseline for inotropic ((c); FOC) and lusitropic ((d); time to 90% relaxation) response. Data are expressed as mean ± 95% CI. Numbers above error bars indicate *p* values <0.05 vs. HLHS (1), TOF (2), 9 wk hiPS-CBs (3), and 16 wk hiPS-CBs (4). Effect on FOC: ANOVA, *p* = 5.06 × 10^−4^; Games-Howell post hoc: HLHS vs. TOF: *p* = 1.00; HLHS vs. hiPS-CBs 9 wk: *p* = 0.057; HLHS vs. hiPS-CBs 16 wk: *p* = 0.067; TOF vs. hiPS-CBs 9 wk: *p* = 2.89 × 10^−3^; TOF vs. hiPS-CBs 16 wk: *p* = 3.95 × 10^−3^; and hiPS-CBs 9 wk vs. hiPS-CBs 16 wk: *p* = 0.085. Effect on time to 90% relaxation: ANOVA, *p* = 5.58 × 10^−6^, Hochberg post hoc: HLHS vs. TOF: *p* = 1.00; HLHS vs. hiPS-CBs 9 wk: *p* = 1.15 × 10^−3^; HLHS vs. hiPS-CBs 16 wk: *p* = 1.04 × 10^−4^; TOF vs. hiPS-CBs 9 wk: *p* = 1.54 × 10^−4^; TOF vs. hiPS-CBs 16 wk: *p* = 1.04 × 10^−4^; and hiPS-CBs 9 wk vs. hiPS-CBs 16 wk: *p* = 1.00.

HLHS and TOF tissue slices showed a positive inotropic response to rising concentrations of ISO. In almost all HLHS tissue slices, increase of force of contraction was first observable at 10^−7^ mol/L ISO, while TOF tissue slices showed a high variability for the onset of positive inotropy (Figure 6(a)). In contrast, 9 wk hiPS-CBs revealed a dose-dependent negative inotropic response and 16 wk hiPS-CBs did not reveal an ascertainable response to ISO except for two preparations that showed a slight increase in force of contraction at higher concentrations (Figure 6(a)).

The contraction and relaxation kinetics were accelerated at higher ISO concentrations in all groups. Time to peak and time of 50% relaxation and 90% relaxation exhibited a dose-dependent decline which was more pronounced in human myocardial tissue groups compared to both hiPS-CB groups (Figure 6(b); Supplemental Table 4; Supplemental Figure 4).

To compare the strength of the ISO effects between the examined groups, the percentage of force at 10^−6^ mol/L ISO relative to the baseline was determined. Consistent with the observed changes of developed force in single experiments, the effect of strength on force of contraction points towards prominent differences in the inotropic response between human myocardial tissue groups and hiPS-CB groups. Force of contraction increased in average 403% and 378% for HLHS and TOF myocardial slices, respectively, while 9 wk hiPS-CBs exhibited a decrease of 17% and 16 wk hiPS-CBs showed a negligible response of 1% (Figure 6(c)). In terms of lusitropy, HLHS and TOF tissue slices demonstrated a considerable decrease of ~24% in time to 90% relaxation while the reduction in both hiPS-CB groups was 3 times less (~8%) (Figure 6(d)).

## 4. Discussion

Inability of pluripotent stem cell-derived cardiomyocytes to adequately react to pharmacological stimuli is often considered as failure to mature under given culture conditions. We thought it was worth asking whether hiPS-CBs might be able to mimic pharmacologic responses of the less mature myocardium. Therefore, the aim of the present study was to compare human iPS-CBs and the neonatal and infantile myocardium with regard to contractile and electrophysiological properties relevant for pharmacological testing.

Up to now, there are only very limited data available on contractile and electrophysiological function of the human myocardium or cardiomyocytes isolated from neonatal or infantile heart. Similarly to our TOF study group, in previous studies tissue was obtained from RVOT of TOF patients in most instances [13–18]; however, as age at surgical TOF repair varies according to local practices, patients are relatively heterogeneous with regard to age. In our institution, TOF surgery is usually scheduled at 6 months of age resulting in a relatively homogenous TOF group in this report. To our knowledge, there is only one report comparing contractile function of neonatal and infantile human myocardial tissue [17]. We are not aware of any published data on electrophysiological properties of human neonatal myocardial tissue at all.

With regard to the parameters investigated, both neonatal and infantile myocardial tissue slices show a behaviour closely similar to the adult myocardium. This is in contrast with the – to our knowledge – only study on contractile properties of myocardial tissue obtained from neonatal patients [17]. Here, a negative FFR was observed in the HLHS myocardium, while in older infants with TOF, FFR was positive. This was interpreted as a developmental effect. In the frequency range used in their study (0.5-2 Hz), we observed a positive FFR in almost all HLHS patients and a varying FFR in our TOF cohort. We cannot confirm the argument that the observed transition from a blunted FFR in neonates to a positive FFR in infants is a developmental effect. This is in line with other mammals, e.g., neonatal mouse [19] or pig [20], which show a positive FFR already early after birth.

As an alternative explanation for observed differences, our data rather suggest that the differences between the two groups might be rather disease-related. Pressure overload and RVOT hypertrophy as it occurred in our TOF patients might result in higher occurrence of impaired FFR. This was observed in a feline model of pulmonary artery banding and attributed to reduced sarcoplasmic reticulum Ca^2+^ load [21]. Furthermore, it was shown that the RVOT of TOF patients exhibits several histopathological features which vary largely between patients [22, 23]. Therefore, the higher variability of observed FFR types in our TOF slices might reflect these disease-related manifestations.

Our results are also conflicting with earlier studies with regard to electrophysiological properties. In a series of reports, Koidl and coworkers found a consistently long APD between 800 ms [16] and 850 ms [15] and spontaneous EADs in cardiomyocytes isolated from RVOT of TOF patients. It was speculated that these remarkably prolonged APDs might contribute to the higher incidence of ventricular arrhythmias in older TOF patients resulting in an increased rate of sudden cardiac death [24]. APD in our study was much shorter in both TOF and HLHS tissue slices and more consistent with APDs reported for human adult cardiomyocytes as well as with another report on infantile cardiomyocytes [13]. One of the main differences between our and the studies of Koidl's group is that children in their study were much older (16-91 months, mean 30 months [16], 16-91 months, mean 35 months [15]) at the time of surgical correction. The observed differences in APD might be a result of the longer duration of RV pressure overload and might be a factor contributing to a reduced arrhythmia load when TOF is repaired early [25].

Comparing the functional characteristics of native ventricular tissue slices and hiPS-CBs in our study, we found (1) a different force-frequency relationship in hiPS-CBs and human heart slices, (2) a blunted inotropic response to *ß*-adrenergic stimulation in hiPS-CBs, and (3) differences in the inducibility of EADs between hiPS-CBs and human tissue slices after I_k,r_ blockade.

Both HLHS and TOF tissue slices showed a dose-dependent positive inotropic response to ISO. Interestingly, TOF tissue slices showed a higher variability of their sensitivity to ISO compared to HLHS slices. A positive inotropic response to ISO has been reported for human foetal heart tissue already at 9 weeks after gestation [26]. We can only speculate why hiPS-CBs fail to show a strong response to ISO, but previous studies found lower expressions of beta-adrenergic receptors [11] and certain key proteins of the calcium-handling machinery [27] in hiPS-CMs compared to the adult human myocardium. While in our study the inotropic state of iPS-CMs was resistant to *ß*-adrenergic stimulation in our study, Germanguz et al. [28] found an increase of contraction up to ~180% in hiPS-CMs after 18-70 days of differentiation and ~130% after 10-15 days of differentiation. This increase is still much lower than in the native ventricular tissue in our study. These data are generated by videographically estimating fractional shortening and are only indirectly related to force of contraction. Therefore, a direct comparison with data from our study is illegitimate.

The same study [28] reported a negative FFR within a frequency range between 0.5 and 1.2 Hz in 10-70 days old iPS-CMs. In our study, FFR turns negative only above 2 Hz both in hiPS-CBs. A positive force-frequency relationship has been suggested as a marker for advanced maturation; it must however be kept in mind that a positive FFR was observed in the human foetal myocardium as early as 9-10 weeks after gestation [26]. Moreover, we could not investigate the FFR over the full range of tested frequencies due to the intrinsic automaticity of hiPS-CBs. Despite that this commonly observed automaticity generally argues for the immaturity of pluripotent stem cell-derived cardiomyocytes, we suggest that the comparison of electrophysiological and contractile properties is still relevant since maturation of individual properties might be independent from each other in an in vitro system.

Given the E4031-induced action potential prolongation, hiPS-CBs showed a positive response to one of the main targets for cardiac safety screening; i.e., they were responsive to I_k,r_ blockade. This has already extensively been shown for iPSC-CMs [29–31]. Our data however highlight two points of major concern when using this technology. Despite an increase in APD, first the extent of APD prolongation was much more pronounced in native tissue slices, and secondly, iPS-CBs were however not able to reproduce afterdepolarisations that occurred in HLHS and TOF tissue slices at the same drug concentrations. A major requirement for screening assays, however, is a high sensitivity. If the effects in the screening assay are less pronounced than in native tissue (AP prolongation) or even absent (induction of EADs), the putative screening assay fails at this criterion.

With the present study, we are able to extend the concern that human iPS-CMs do not represent the adult cardiomyocyte physiology well enough to be useful for cardiotoxicity screening [32] to the less mature neonatal and infantile myocardium.

Promising improvements in addition to prolonged culture times have been made in driving maturation of pluripotent stem cell-derived cardiomyocytes [9]. This might lead to improvements in cardiac safety assays in the future; however, a preparation- and age-specific comparison of pharmacological effects between native and stem cell-derived CMs will be warranted.

### 4.1. Limitations

The main aims of our study were to test the effects of specific interventions on physiological functions such as the effect of beta-adrenergic stimulation on contractile force. As we have not normalised physiological parameters to properties of individual preparations (e.g., cross-sectional area of tissue slices or cell number), comparability between measurements remains impaired.

A major limitation of this study is the comparison of iPSC-CBs derived from healthy human donors and ventricular tissue slices obtained from patients with cyanotic congenital heart disease. Tissue from healthy human donors of this age group is simply not available. Nevertheless, we think that our findings can recapitulate the key hallmarks of electrophysiological and contractile properties of the young human myocardium.

## Figures and Tables

**Table 1:** Patient characteristics.

Patient ID	Age (days/months)	Birth weight (g)	Diagnosis	Type of operation	Drugs	SpO_2_	Experiments
HLHS1	36/1.2	3250	HLHSMSAS	Norwood procedure	M, D	99	FFR
HLHS2	15/0.5	2690	HIAA + VSD	Norwood procedure	M, BB, F, S, D	90	RDA, E4031, FFR, ISO
HLHS3	69/2.3	2100	HLHS	Norwood procedure	BB, ASA, D	70	RDA
HLHS4	12/0.4	3900	HLHSMA + TAPVR	Norwood procedure	M, Mil, D	95	RDA, E4031, FFR, ISO
HLHS5	6/0.2	2570	HLHSMAAA	Norwood procedure	M	95	RDA, FFR, ISO
HLHS6	7/0.2	2800	HLHS	Norwood procedure	M, BB, S, D	95	RDA, FFR, ISO
HLHS7	2/0.1	2700	HLHS + MS + AS	Norwood procedure	M, D	90	RDA, FFR, ISO
TOF1	168/5.5	3950	TOF + PDA	TOF repair	BB	91	RDA, FFR, ISO
TOF2	170/5.6	3000	TOF	TOF repair	Nil	99	RDA, E4031
TOF3	198/6.5	3200	TOF	TOF repair	Sot	100	RDA, E4031, FFR, ISO
TOF4	164/5.4	3300	TOF	TOF repair	A, S, BB, ASA	72	FFR, ISO
TOF5	210/6.9	3850	PA + VSD	VSD closure, RV-PA-conduit	ASA, BB	80	RDA, E4031 + ISO, FFR, ISO
TOF6	178/5.8	1550	TOF + PDA	TOF repair, closure of PDA	BB	65	RDA, E4031 + ISO, FFR, ISO
TOF7	129/4.2	3250	TOF	TOF repair	Nil	99	RDA, E4031 + ISO, FFR
TOF8	155/5.1	3100	TOF	TOF repair	Nil	98	FFR
TOF9	212/7.0	2650	PA-VSD	VSD closure, RV-PA conduit	ASA	75	RDA, FFR
TOF10	165/5.5	2900	TOF	TOF repair	Nil	92	E4031

M: male; F: female; AS: aortic stenosis; IAA: interrupted aortic arch; HLHS: hypoplastic left heart syndrome; PA: pulmonary atresia; PDA: patent ductus arteriosus; TAPVR: total anomalous pulmonary venous return; TOF: tetralogy of Fallot; VSD: ventricular septal defect; A: ACE inhibitor; ASA: acetylsalicylic acid; BB: *ß*-blocker; D: dexamethasone; F: furosemide; P: prostaglandin E2; Mil: milrinone; S: spironolactone; Sot: sotalol; oxyhemoglobin saturation by pulse oximetry; FFR: force-frequency relationship; ISO: isoproterenol; RDA: rate-dependent action potential adaptation.

## Data Availability

Data are available from the authors upon request.

## Supplementary Materials

Supplementary Materials Supplementary Materials: Suppl_Figure_1_exp_overview.eps; Supplemental Figure 1: experimental overview for generation and measurements of human myocardial tissue biopsies and hiPS-CBs. Suppl_Figure_2_FDAR.eps; Supplemental Figure 2: analysis of frequency-dependent acceleration of relaxation (FDAR). Suppl_Figure_3_FFR.eps; Supplemental Figure 3: representative traces of force-frequency relationships (FFR) pattern. Suppl_Figure_4_ISO.eps; Supplemental Figure 4: representative traces of experiments before and after *ß*-adrenergic stimulation by isoproterenol. Trieschmann_Supp_Table1.xlsx; Supplemental Table 1: descriptive and inferential statistics of action potential (AP) parameters. Trieschmann_Supp_Table2.xlsx; Supplemental Table 2: descriptive and inferential statistics of action potential (AP) parameters for I_k,r_ blockade experiments. Trieschmann_Supp_Table3.xlsx Supplemental Table 3: descriptive and inferential statistics for force-frequency relationship (FFR) experiments. Trieschmann_Supp_Table4.xlsx; Supplemental Table 4: descriptive and inferential statistics of twitch morphology for *ß*-adrenergic stimulation experiments.
