# Supplementary material for: Different Responses to Drug Safety Screening Targets between Human Neonatal and Infantile Heart Tissue and Cardiac Bodies Derived from Human-Induced Pluripotent Stem Cells
Source: Stem Cells Int. 2019 Mar 6;2019:6096294. doi: 10.1155/2019/6096294 (PMC6431377; doi:10.1155/2019/6096294)
Supplement: Supplementary Materials — Suppl_Figure_1_exp_overview.eps; Supplemental Figure 1: experimental overview for generation and measurements of human myocardial tissue biopsies and hiPS-CBs. Suppl_Figure_2_FDAR.eps; Supplemental Figure 2: analysis of frequency-dependent acceleration of relaxation (FDAR). Suppl_Figure_3_FFR.eps; Supplemental Figure 3: representative traces of force-frequency relationships (FFR) pattern. Suppl_Figure_4_ISO.eps; Supplemental Figure 4: representative traces of experiments before and after ß-adrenergic stimulation by isoproterenol. Trieschmann_Supp_Table1.xlsx; Supplemental Table 1: descriptive and inferential statistics of action potential (AP) parameters. Trieschmann_Supp_Table2.xlsx; Supplemental Table 2: descriptive and inferential statistics of action potential (AP) parameters for Ik,r blockade experiments. Trieschmann_Supp_Table3.xlsx Supplemental Table 3: descriptive and inferential statistics for force-frequency relationship (FFR) experiments. Trieschmann_Supp_Table4.xlsx; Supplemental Table 4: descriptive and inferential statistics of twitch morphology for ß-adrenergic stimulation experiments. [file 6096294.f1.pdf]

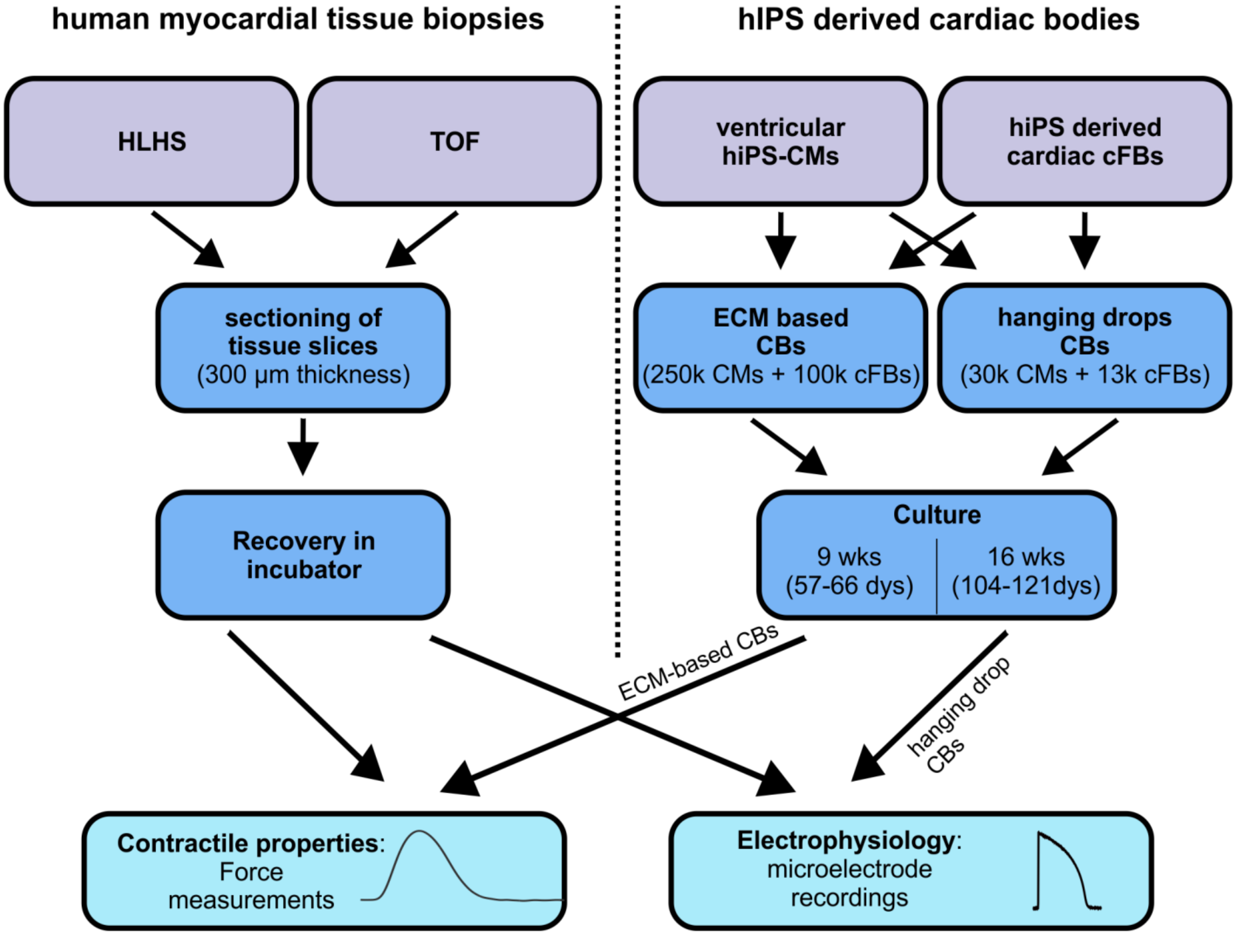

Supplemental Fig 1: Experimental overview. Human myocardial tissue biopsies from HLHS and TOF patients are sectioned before electrophysiological recordings and force measurements on the same day. Cardiac bodies (CBs) were produced from hiPS derived ventricular cardiomyocytes and cardiac fibroblasts and cultured until experiments. Microelectrode recordings were performed with CBs formed by hanging drop method, while force measurements were carried out with CBs produced by an extracellular matrix (ECM) based approach.

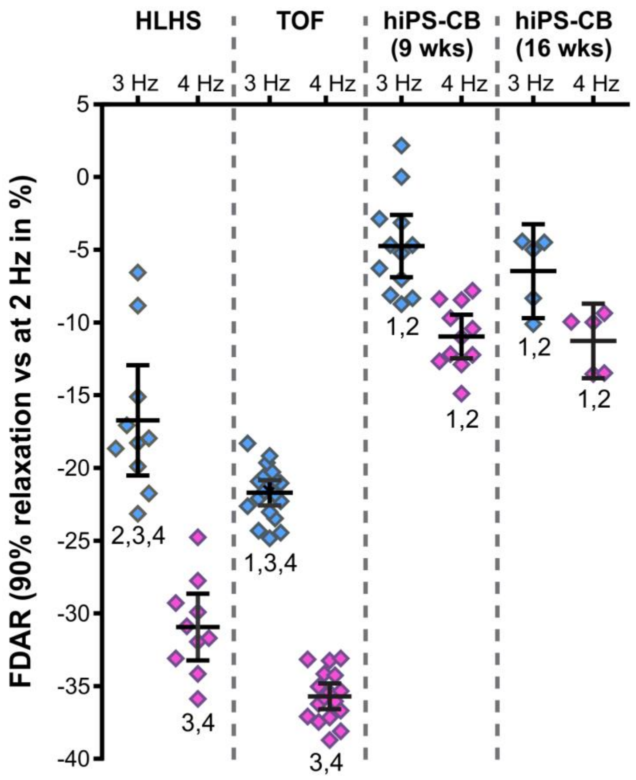

Supplemental Fig 2: Frequency-dependent acceleration of relaxation (FDAR). All groups show FDAR at a stimulation frequency of 3 Hz and 4Hz as indicated by the reduction in time to 90% relaxation relative to contractions at 2 Hz of individual experiments (HLHS, n = 10 of 6 patients; TOF, n = 19 of 8 patients; 9 wks hIPS-CBs, n = 12; 16 wks hiPS-CBs, n = 5). Lines and error bars represent mean ± 95% CIs, respectively. Numbers below error bars indicate p-values < 0.05 vs. HLHS (1), TOF (2), 9 wks hiPS-CBs (3), and 16 wks hiPS-CBs (4). 3Hz: ANOVA, p=5.99 x 10^{-28}; Games Howell Post-Hoc: HLHS vs. TOF: p = 0.004; HLHS vs. hiPS-CBs 9 wks: p = 1.29 x 10^{-10}; HLHS vs. hIPS-CBs 16 wks: p =4.04 x 10^{-8} ; TOF vs. hiPS-CBs 9 wks: p =1.07 x 10^{-12}; TOF vs. hiPS-CBs 16 wks: p = 2.20 x 10^{-6}; hiPS-CBs 9 wks vs. hiPS-CBs 16 wks: p = 0.994. 4Hz: ANOVA, p=1.39 x 10^{-9}; Games Howell Post-Hoc: HLHS vs. TOF: p = 0.065; HLHS vs. hiPS-CBs 9 wks: p = 1.00 x 10^{-4}; HLHS vs. hIPS-CBs 16 wks: p = 1.15 x 10^{-3}; TOF vs. hiPS-CBs 9 wks: p = 3.99 x 10^{-10}; TOF vs. hiPS-CBs 16 wks: p = 2.24 x 10^{-4}; hiPS-CBs 9 wks vs. hiPS-CBs 16 wks: p = 0.676.

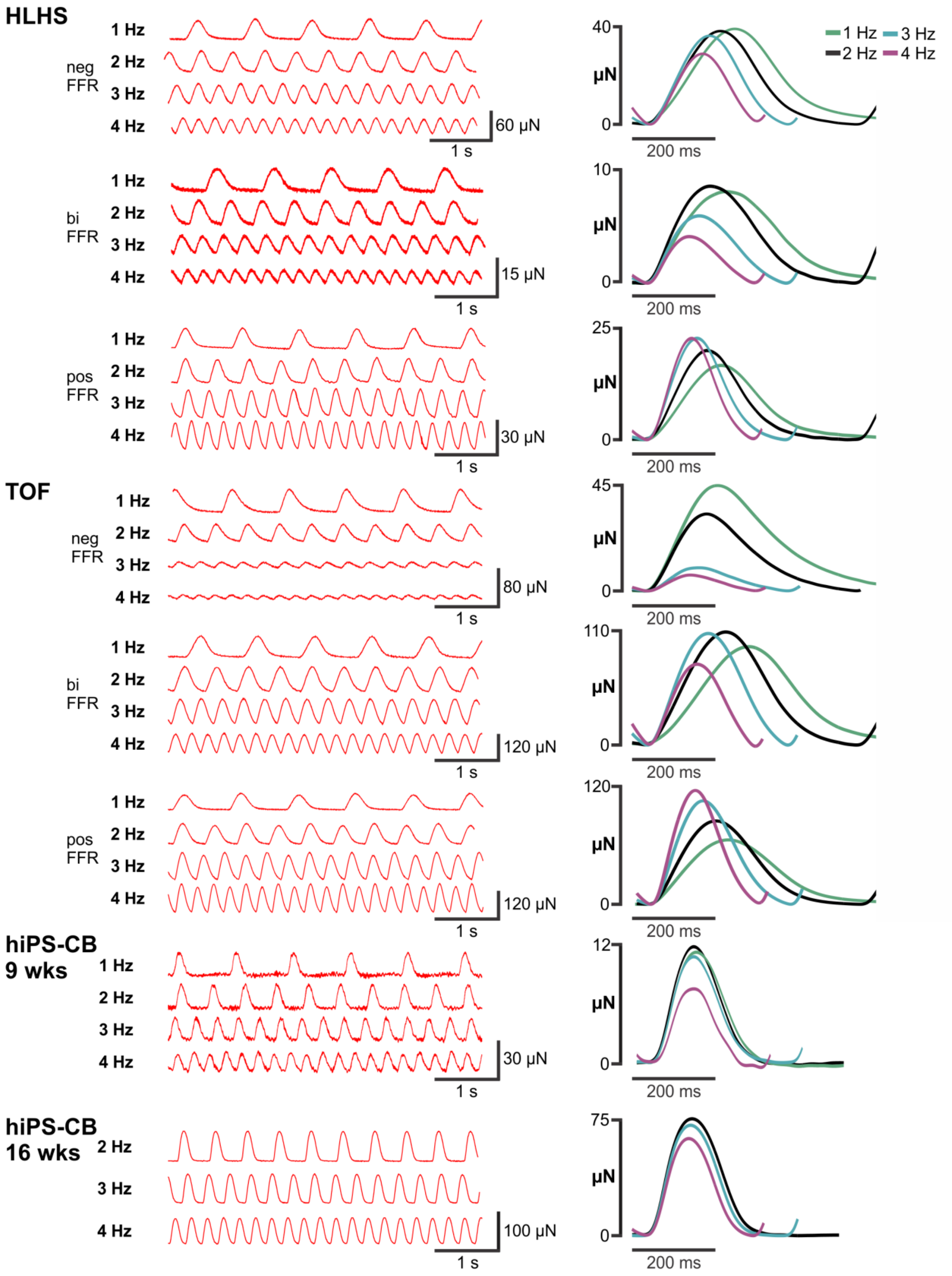

Supplemental Fig 3: Representative experiments of force-frequency relationships (FFR) pattern. For HLHS and TOF myocardial tissues slices, examples are shown for positive (HLHS6, TOF3), negative (HLHS6, TOF9) and bi-phasic FFRs (HLHS2 , TOF4). Because hiPS-CBs did not exhibit diverse FFR patterns, only one representative experiment for 9 wks and 16 wks is depicted. The red traces on the left side show original recordings of

experiments at different stimulation frequencies (1,2,3,4 Hz) and the corresponding averaged twitches are superimposed in the right panels. Note that none of the 16 wks old hiPS-CBs could be stimulated at 1 Hz.

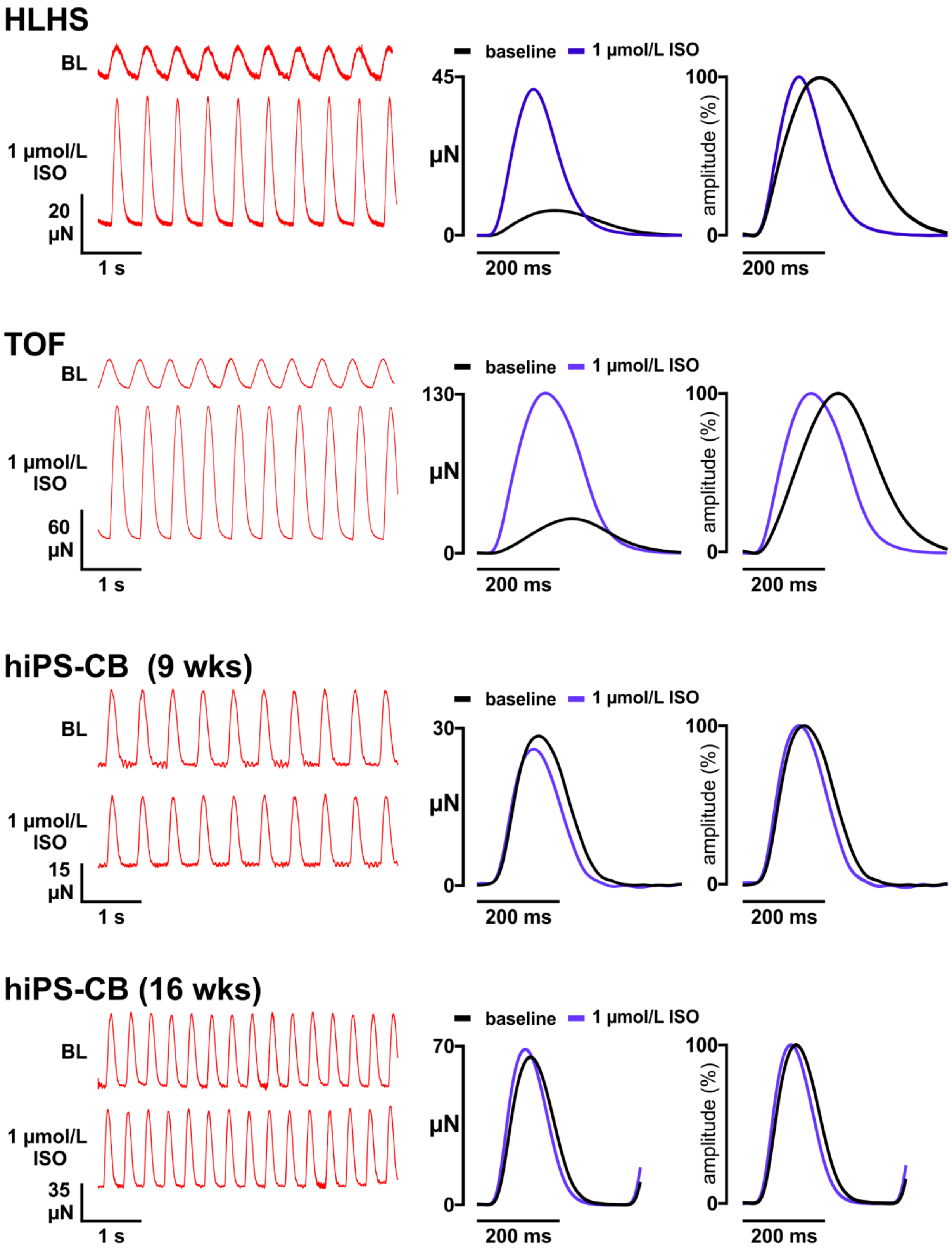

Supplemental Fig 4: Representative experiments of ß-adrenergic stimulation by isoproterenol. Original traces (left panels), averaged twitches (middle panels) and amplitude-normalized averaged twitches (right panels) before and after application of 1 µmol/L isoproterenol (ISO) for myocardial tissues slices from patients HLHS2 and TOF4 as well as hiPS-CBs (9 wks and 16 wks) are depicted. Lusitropic effect of ß-adrenergic stimulations is highlighted by amplitude normalisation of the averaged twitches.

<u>Tables:</u>

Table 1: Patients characteristics.

Patient ID	Age (days/months)	Birth weight (g)	Diagnosis[2]	Type of operation	Drugs[3]	SpO_2 [4]	Experiments[5]
HLHS1	36 / 1.2	3250	HLHSMSAS	Norwood procedure	M, D	99	FFR
HLHS2	15 / 0.5	2690	HIAA+VSD	Norwood procedure	M, BB, F, S, D	90	RDA,E4031,FFR, ISO
HLHS3	69 / 2.3	2100	HLHS	Norwood procedure	BB, ASA, D	70	RDA
HLHS4	12 / 0.4	3900	HLHSMA+TAPVR	Norwood procedure	M, Mil, D	95	RDA,E4031, FFR, ISO
HLHS5	6 / 0.2	2570	HLHSMAAA	Norwood procedure	M	95	RDA, FFR, ISO
HLHS6	7 / 0.2	2800	HLHS	Norwood procedure	M, BB, S, D	95	RDA, FFR, ISO
HLHS7	2 / 0.1	2700	HLHS+MS+AS	Norwood procedure	M, D	90	RDA, FFR, ISO
TOF1	168 / 5.5	3950	TOF+PDA	TOF repair	BB	91	RDA, FFR, ISO
TOF2	170 / 5.6	3000	TOF	TOF repair	Nil	99	RDA,E4031
TOF3	198 / 6.5	3200	TOF	TOF repair	Sot	100	RDA,E4031, FFR, ISO
TOF4	164 / 5.4	3300	TOF	TOF repair	A, S, BB, ASA	72	FFR, ISO
TOF5	210 / 6.9	3850	PA+VSD	VSD closure, RV-PA-conduit	ASA, BB	80	RDA,E4031+ISO, FFR, ISO
TOF6	178 / 5.8	1550	TOF+PDA	TOF repair, closure of PDA	BB	65	RDA,E4031+ISO, FFR, ISO
TOF7	129 / 4.2	3250	TOF	TOF repair	Nil	99	RDA,E4031+ISO, FFR
TOF8	155 / 5.1	3100	TOF	TOF repair	Nil	98	FFR
TOF9	212 / 7.0	2650	PA-VSD	VSD closure, RV-PA-conduit	ASA	75	RDA, FFR
TOF10	165 / 5.5	2900	TOF	TOF repair	Nil	92	E4031

1: M, male; F, female

2: AS, aortic stenosis; IAA, interrupted aortic arch; HLHS, hypoplastic left heart syndrome; PA, pulmonary atresia; PDA, patent ductus arteriosus; TAPVR, total anomalous pulmonary venous return; TOF, Tetralogy of Fallot; VSD, ventricular septal defect

3: A, ACE-inhibitor; ASA, acetylsalicylic acid; BB, ß-blocker; D, dexamethasone; F, furosemide ; P, prostaglandin E2; Mil, milrinone; S, spironolactone ; Sot, sotalol

4: Oxyhemoglobin saturation by pulse oximetry

5: FFR, force-frequency-relationship; ISO, isoproterenol; RDA, rate-dependent action potential adaptation.

Supplemental Table 1: AP characteristics.

	frequency		amplitude			MDP			Vmax			APD 20			APD 50			APD 90		
			mean	95% CI	p-value vs. 2 Hz	mean	95% CI	p-value vs. 2 Hz	mean	95% CI	p-value vs. 2 Hz	mean	95% CI	p-value vs. 2 Hz	mean	95% CI	p-value vs. 2 Hz	mean	95% CI	p-value vs. 2 Hz
	Hz	n	mV	mV		ms	ms		mV/s	mV/s		ms	ms		ms	ms		ms	ms	
**HLHS**				RM: 1.89×10^{-4}			RM: 3.13×10^{-3}			RM: 4.65×10^{-3}			RM: 1.65×10^{-12}			RM: 1.73×10^{-13}			RM: 7.34×10^{-14}	
	1	16	108.6	[104.2, 113.1]	0.017	-81.0	[-85.4, -76.6]	0.029	155.0	[111.8, 198.3]	0.032	126.1	[111.6, 140.5]	1.84×10^{-5}	245.2	[225.0, 265.4]	1.59×10^{-4}	327.8	[308.8, 346.8]	1.68×10^{-4}
	2	16	106.3	[102.2, 110.4]	-	-78.1	[-81.7, -74.5]	-	146.0	[110.3, 181.6]	-	105.3	[91.7, 118.8]	-	212.3	[196.5, 228.1]	-	291.4	[278.4, 304.3]	-
	3	16	103.5	[98.1, 108.9]	7.21×10^{-4}	-76.6	[-79.9, -73.3]	0.11	133.9	[92.0, 175.8]	0.1	83.0	[74.4, 91.7]	1.84×10^{-5}	166.3	[155.9, 176.6]	3.00×10^{-9}	240.2	[231.2, 249.3]	1.85×10^{-9}
**TOF**				RM: 1.58×10^{-3}			RM:0.21			RM: 0.022			FA: 4.59×10^{-10}			RM: 5.84×10^{-11}			RM: 1.51×10^{-9}	
	1	17	107.9	[103.7, 112.1]	0.82	-81.3	[-85.3, -77.3]	0.81	133.9	[113.7, 154.2]	0.46	135.2	[118.1, 152.3]	4.38×10^{-4}	264.5	[203.5, 298.5]	2.23×10^{-5}	360.1	[320.6, 399.6]	2.35×10^{-5}
	2	17	107.4	[102.4, 112.5]	-	-80.0	[-84.4, -75.7]	-	126.1	[102.7, 149.4]	-	119.6	[103.2, 135.9]	-	231.0	[203.0, 259.0]	-	319.0	[287.9, 350.0]	-
	3	13	101.1	[94.5, 107.6]	4.21×10^{-3}	-75.8	[-81.4, -70.1]	0.15	112.6	[90.7, 134.5]	1.72×10^{-3}	93.9	[82.3, 105.5]	3.59×10^{-5}	181.9	[163.0, 200.6]	5.75×10^{-7}	265.6	[241.0, 290.3]	1.82×10^{-6}
**iPS-CBs, 9 wks**				RM: 0.030			RM: 0.56			RM: 0.70			RM: 0.045			RM: 5.96 x 10-3			RM: 0.066	
	1	9	106.0	[102.1, 109.8]	0.1	-75.1	[-78.3, -72.0]	0.42	90.6	[55.4, 124.8]	0.67	75.0	[70.0, 80.0]	0.38	138.6	[130.1, 147.2]	8.25×10^{-3}	189.6	[176.7, 202.5]	0.077
	2	17	106.0	[103.1, 108.9]	-	-73.8	[-76.4, -71.2]	-	88.9	[65.2, 112.7]	-	68.8	[64.8, 72.8]	-	123.9	[118.9, 129.0]	-	176.1	[167.8, 184.5]	-
	3	12	104.6	[100.7, 108.4]	0.019	-73.9	[-77.7, -70.1]	0.96	80.0	[54.6, 105.3]	0.22	63.9	[59.7, 68.1]	0.086	117.6	[112.3, 123.0]	9.18×10^{-3}	170.1	[161.3, 179.0]	0.07
**iPS-CBs, 16 wks**				RM: 0.19			RM: 0.77			n.a.			RM: 0.12			RM: 0.19			RM: 0.19	
	1	4	96.0	[86.7, 105.4]	0.29	-62.1	[-69.1, -55.1]	0.64	n.a.	n.a.	n.a.	62.4	[55.2, 69.6]	0.26	116.5	[99.2, 133.8]	0.36	173.5	[142.5, 204.6]	0.33
	2	10	94.0	[89.9, 98.2]	-	-62.3	[-67.1, -57.5]	-	45.7	[n=2]	-	53.8	[49.2, 58.4]	-	101.3	[93.7, 109.0]	-	149.0	[138.8, 159.3]	-
	3	5	95.5	[88.3, 102.7]	0.26	-61.9	[-71.7, -52.1]	0.037	46.5	[21.4, 71.6]	n.a.	51.6	[46.2, 57.0]	0.15	94.2	[85.2, 103.3]	0.22	139.4	[129.1, 149.8]	0.19

RM, ANOVA repeated measures & LSD Post-Hoc testing

FA, Friedman ANOVA & Wilcoxon pairwise testing

1, p-values are computed for RM/FA and individual groups compared to 2Hz

n.a. indicates missing data/lack of post-hoc testing

Supplemental Table 2: Effects of $I_{K,r}$ blockade.

			amplitude			MDP			Vmax			APD 50			APD 90			EAD
			mean	95% CI	p-value vs. BL (*E4031)	mean	95% CI	p-value vs. BL (*E4031)	mean	95% CI	p-value vs. BL (*E4031)	mean	95% CI	p-value vs. BL (*E4031)	mean	95% CI	p-value vs. BL (*E4031)	
		n	mV	mV		ms	ms		mV/s	mV/s		ms	ms		ms	ms		
**HLHS**																		
	Baseline	3	109.1	[78.5, 139.6]	-	-80.0	[-96.8, -63.1]	-	149.4	[-74.4, 373.1]	-	225.2	[197.1, 253.3]	-	302.8	[279.8, 325.8]	-	0/3
	E4031 1µM	3	114.9	[111.7, 118.0]	0.52	-85.1	[-97.1, -73.0]	0.75	192.3	[88.29, 296.2]	0.33	352.4	[252.1, 452.6]	0.047	489.9	[342.2, 637.6]	0.032	2/3
**Fallot**																		
	Baseline	6	108.9	[104.4, 113.4]	-	-76.5	[-85.9, -66.5]	-	120.2	[92.2, 148.1]	-	263.5	[182.2, 344.9]	-	327.4	[244.1, 410.7]	-	0/6
	E4031 1µM	6	101.9	[91.9, 112.0]	0.11	-71.6	[-82.7, -60.6]	0.23	97.7	[85.6, 109.7]	0.037	345.0	[259.9, 430.1]	$1,31 \times 10^{-3}$	477.6	[365.6, 589.6]	$5,53 \times 10^{-4}$	2/6
**iPS-CBs, 9 wks**																		
	Baseline	7	106.2	[102.1, 110.3]	-	-78.1	[-82.5, -73.7]	-	96.5	[48.5, 144.5]	-	124.2	[114.4, 134.0]	-	176.7	[156.1, 197.3]	-	0/7
	E4031 1µM	7	106.3	[100.1, 112.5]	0.97	-74.6	[-77.6, -71.7]	0.28	68.2	[33.3, 103.1]	0.21	146.1	[133.9, 158.2]	$6,46 \times 10^{-3}$	248.5	[225.2, 271.8]	$3,51 \times 10^{-3}$	0/7
**Fallot**				RM: 0.32			RM: 0.14			n.a.			RM: 0.043			RM: 0.021		
	Baseline	7	104.4	[98.2, 110.7]	-	-75.1	[-78.6, -71.52]	-	128.1	[95.1, 161.0]	-	220.6	[193.2, 247.9]	-	335.8	[308.4, 363.3]	-	
	E4031 0.1µM	7	100.9	[91.4, 110.3]	0.45	-74.1	[-78.3, -69.9]	0.33	78.7	[62.7, 94.7]	n.a.	293.7	[252.9, 334.5]	2.32×10^{-4}	511.9	[442.7, 581.0]	2.15×10^{-3}	
	+Isoproterenol	5	110.1	[104.5, 115.8]	0.23*	-81.0	[-85.9, -76.2]	6.56×10^{-3}*	56.2	[n.a.]	n.a.	270.0	[190.3, 349.7]	0.27*	463.4	[291.4, 635.4]	0.38*	
**iPS-CBs, 9 wks**				RM: 3.78×10^{-3}			RM: 0.88			RM: 0.66			FA: 0.050			RM: 0.067		
	Baseline	8	105.5	[98.7, 112.3]	-	-74.2	[-77.2, -71.1]	-	86.5	[47.4, 125.7]	-	122.1	[115.3, 128.8]	-	168.5	[162.5, 174.5]	-	
	E4031 0.1µM	8	110.8	[106.8, 114.7]	0.037	-73.4	[-97.5, -49.2]	0.77	98.2	[75.2, 121.3]	0.53	130.0	[121.9, 138.1]	0.076	213.0	[195.3, 230.7]	3.14×10^{-3}	
	+Isoproterenol	7	110.7	[106.3, 115.1]	0.26*	-75.5	[-86.2, -64.8]	0.97*	87.6	[70.3, 104.9]	0.49*	111.3	[80.2, 142.5]	0.18*	191.8	[152.5, 231.0]	0.40*	
**iPS-CBs, 16 wks**				RM: 0.55			RM: 0.48			RM: 0.63			RM: 0.24			RM: 0.033		
	Baseline	6	95.3	[86.7, 103.9]	-	-70.4	[-75.2, -65.6]	-	18.0	[3.1, 33.0]	-	106.2	[84.5, 127.9]	-	148.1	[117.8, 178.3]	-	
	E4031 0.1µM	5	97.0	[89.6, 104.4]	0.45	-67.2	[-92.1, -42.3]	0.61	14.4	[11.9, 16.9]	0.85	133.3	[115.4, 151.2]	0.12	239.0	[219.5, 258.6]	0.048	
	+Isoproterenol	4	91.9	[84.8, 98.9]	0.83*	-60.2	[-80.3, -40.2]	0.43*	16.6	[9.9, 23.4]	0.45*	101.0	[64.2, 137.7]	0.17*	181.7	[126.3, 237.1]	0.042*	

BL, baseline

RM, ANOVA repeated measures & LSD Post-Hoc testing

FA, Friedman ANOVA & Wilcoxon pairwise testing

1, p-values are computed for RM/FA and individual groups compared to baseline or E4031 (*)

n.a. indicates missing data/lack of post-hoc testing

Supplemental Table 3: Force Frequency Relationship (FFR).

	Hz	n	time to peak mean (ms)	time to peak 95% CI (ms)	time to peak p-value[1] vs. 2 Hz	time to 50% relaxation mean (ms)	time to 50% relaxation 95% CI (ms)	time to 50% relaxation p-value[1] vs. 2 Hz	time to 90% relaxation mean (ms)	time to 90% relaxation 95% CI (ms)	time to 90% relaxation p-value[1] vs. 2 Hz
**HLHS**				FA: 2.81×10^{-22}			FA: 1.67×10^{-23}			RM: 9.90×10^{-11}	
	0.5	9	226.7	[205, 248]	3.91×10^{-3}	348.2	[323, 374]	3.91×10^{-3}	475.2	[433, 518]	3.49×10^{-5}
	1	10	208.7	[191, 227]	3.91×10^{-3}	310.4	[285, 336]	3.91×10^{-3}	436.7	[400, 474]	9.63×10^{-4}
	1.5	10	196.5	[184, 209]	1.95×10^{-3}	299.4	[260, 291]	1.95×10^{-3}	417.9	[391, 445]	1.60×10^{-4}
	2	10	182.2	[172, 193]	-	275.3	[260, 291]	-	377.8	[359, 397]	-
	1.5	10	162.8	[153, 173]	1.95×10^{-3}	241.6	[228, 255]	1.95×10^{-3}	317.7	[303, 333]	3.49×10^{-5}
	3	10	166.9	[154, 180]	1.95×10^{-3}	249.1	[231, 268]	1.95×10^{-3}	330.1	[309, 351]	1.25×10^{-5}
	4	10	143.0	[133, 153]	1.95×10^{-3}	208.1	[197, 220]	1.95×10^{-3}	260.3	[252, 268]	9.81×10^{-8}
**TOF**				RM: 7.61×10^{-13}			RM: 4.72×10^{-22}			RM: 1.09×10^{-30}	
	0.5	19	259.3	[245, 274]	2.68×10^{-10}	401.1	[387, 415]	2.48×10^{-17}	552.4	[539, 566]	7.41×10^{-15}
	1	19	240.1	[230, 251]	2.25×10^{-10}	368.1	[357, 379]	1.29×10^{-11}	514.2	[502, 527]	2.43×10^{-14}
	1.5	19	224.2	[216, 232]	5.78×10^{-11}	345.1	[336, 354]	2.21×10^{-11}	482.3	[471, 494]	6.64×10^{-14}
	2	19	204.5	[197, 212]	-	314.6	[307, 323]	-	423.7	[415, 433]	-
	1.5	19	186.9	[181, 193]	5.33×10^{-8}	286.2	[279, 293]	5.48×10^{-11}	374.4	[368, 381]	1.46×10^{-13}
	3	19	172.8	[167, 179]	5.55×10^{-10}	261.7	[255, 268]	2.82×10^{-13}	331.5	[326, 337]	1.31×10^{-16}
	4	17	153.7	[148, 159]	5.27×10^{-11}	224.1	[218, 231]	9.72×10^{-15}	271.5	[267, 276]	1.03×10^{-18}
**hiPS-CBs, 9 wks**				RM: 1.83×10^{-3}			RM: 2.61×10^{-6}			RM: 2.56×10^{-5}	
	0.5	2	n.a.	n.a.	0.090	n.a.	n.a.	0.031	n.a.	n.a.	1.0
	1	6	143.8	[130, 158]	0.16	228.2	[209, 248]	0.079	300.3	[273, 327]	0.045
	1.5	8	147.0	[138, 156]	0.27	227.0	[215, 239]	0.058	289.4	[274, 304]	0.090
	2	12	140.6	[135, 146]	-	218.3	[210, 227]	-	280.2	[267, 293]	-
	1.5	12	137.3	[132, 143]	0.13	209.3	[201, 217]	0.17	266.7	[255, 278]	0.80
	3	12	140.6	[132, 149]	0.5	212.8	[205, 221]	0.23	272.8	[263, 283]	0.50
	4	11	136.1	[131, 141]	0.5	199.0	[191, 207]	0.084	249.5	[239, 260]	0.028
**hiPS-CBs, 16 wks**				RM: 1.86×10^{-06}			RM: 2.954×10^{-4}			RM: 6.84×10^{-08}	
	0.5	0	n.a.	n.a.	n.a.	n.a.	n.a.	n.a.	n.a.	n.a.	n.a.
	1	0	n.a.	n.a.	n.a.	n.a.	n.a.	n.a.	n.a.	n.a.	n.a.
	1.5	0	n.a.	n.a.	n.a.	n.a.	n.a.	n.a.	n.a.	n.a.	n.a.
	2	5	142.6	[134, 151]	-	218.6	[206, 231]	-	270.6	[258, 284]	-
	1.5	5	136.8	[130, 144]	0.040	206.0	[196, 216]	4.55×10^{-3}	253.0	[241, 265]	2.77×10^{-3}
	3	5	139.6	[134, 145]	1.92×10^{-3}	211.2	[200, 223]	3.33×10^{-3}	259.6	[248, 271]	5.73×10^{-3}
	4	5	133.2	[127, 140]	7.99×10^{-4}	195.8	[187, 205]	5.09×10^{-4}	240.0	[231, 249]	4.41×10^{-4}

RM, ANOVA repeated measures & LSD Post-Hoc testing

FA, Friedman ANOVA & Wilcoxon pairwise testing

1, p-values are computed for RM/FA and individual groups compared to 2Hz

n.a. indicates missing data/lack of post-hoc testing

Supplemental Table 4: Effect of ß-adrenergic stimulation on twitch morphology.

			Relative force (change to BL)			time to peak			time to 50% relaxation			time to 90% relaxation		
			mean	95% CI	p-value[1] vs. 10^{-10} mol/L	mean	95% CI	p-value[1] vs. BL	mean	95% CI	p-value[1] vs. BL	mean	95% CI	p-value[1] vs. BL
	mol/L	n	%	%		ms	ms		ms	ms		ms	ms	
**HLHS**			RM: 5.24×10^{-5}			RM: 7.37×10^{-10}			FA: 1.95×10^{-8}			FA: 2.55×10^{-8}		
	BL	6	0	[0, 0]	-	185	[165, 205]	-	278	[246, 309]	-	374	[341, 407]	-
	10^{-10}	5	-9	[-17, -1]	-	185	[160, 210]	0.32	280	[239, 320]	0.68	180	[331, 420]	0.81
	10^{-9}	6	-12	[-24, -1]	0.44	180	[163, 197]	0.069	273	[247, 300]	0.14	372	[343, 401]	0.53
	10^{-8}	6	-12	[-131, 107]	0.34	166	[143, 188]	0.049	246	[218, 275]	0.028	338	[308, 368]	0.031
	10^{-7}	6	159	[23, 296]	0.052	151	[125, 178]	3.93×10^{-3}	222	[184, 260]	0.028	294	[244, 344]	0.031
	10^{-6}	6	403	[99, 707]	0.032	149	[132, 166]	7.38×10^{-4}	218	[192, 243]	0.027	285	[252, 318]	0.031
	10^{-5}	6	478	[129, 826]	0.028	150	[134, 166]	2.25×10^{-3}	220	[199, 241]	0.028	283	[257, 309]	0.031
**TOF**			FA: 4.46×10^{-4}			RM: 4.63×10^{-5}			RM: 2.10×10^{-4}			RM: 7.09×10^{-5}		
	BL	11	0	[0, 0]	-	201	[188, 214]	-	303	[286, 320]	-	406	[388, 425]	-
	10^{-10}	10	5	[-9, 19]	-	199	[184, 214]	0.64	299	[279, 319]	0.51	402	[378, 425]	1.00
	10^{-9}	11	44	[-26, 113]	0.63	192	[174, 210]	1.00	286	[256, 312]	0.30	383	[348, 419]	0.17
	10^{-8}	11	122	[12, 233]	0.38	176	[160, 192]	0.15	264	[241, 288]	0.13	356	[318, 394]	0.27
	10^{-7}	11	216	[118, 314]	3.91×10^{-3}	164	[156, 172]	0.077	247	[232, 262]	0.096	328	[302, 354]	0.077
	10^{-6}	11	378	[199, 557]	1.95×10^{-3}	155	[148, 162]	0.049	231	[218, 245]	0.063	307	[291, 322]	0.039
	10^{-5}	3	336	[-401, 1072]	0.25	154	[131, 176]	0.048	230	[201, 259]	0.076	312	[284, 341]	0.061
**hiPS-CBs, 9 wks**			FA: 9.03×10^{-4}			RM: 3.68×10^{-8}			RM: 2.09×10^{-6}			RM: 7.38×10^{-6}		
	BL	5	0	[0, 0]	-	141	[127, 155]	-	223	[194, 251]	-	288	[245, 331]	-
	10^{-10}	5	-1	[-8, 7]	-	139	[125, 153]	0.16	223	[199, 247]	0.93	292	[253, 330]	0.57
	10^{-9}	5	-2	[-13, 9]	0.63	140	[125, 156]	0.21	221	[197, 246]	0.35	286	[250, 322]	0.50
	10^{-8}	5	-7	[-19, 6]	0.13	139	[124, 154]	0.12	221	[195, 247]	0.12	285	[248, 322]	0.29
	10^{-7}	5	-15	[-22, -9]	0.063	136	[123, 150]	0.013	217	[194, 240]	0.18	281	[247, 316]	0.20
	10^{-6}	5	-17	[-27, -6]	0.063	127	[118, 137]	7.09×10^{-3}	201	[187, 216]	0.025	263	[237, 289]	0.030
	10^{-5}	5	-21	[-36, -5]	0.063	124	[112, 135]	4.35×10^{-3}	196	[178, 215]	0.030	255	[231, 280]	0.038
**hiPS-CBs, 16 wks**			RM: 0.49			FA: 6.29×10^{-3}			RM: 1.64×10^{-4}			RM: 2.15×10^{-4}		
	BL	5	0	[0, 0]	-	134	[130, 138]	-	200	[192, 208]	-	248	[240, 257]	-
	10^{-10}	5	-2	[-4, -1]	-	134	[131, 194]	1.0	201	[194, 207]	0.76	249	[242, 255]	0.90
	10^{-9}	5	-4	[-6, -2]	n.a.	134	[131, 136]	1.0	199	[193, 206]	0.78	247	[240, 255]	0.76
	10^{-8}	5	-4	[-7, -1]	n.a.	132	[124, 139]	0.75	197	[185, 209]	0.56	244	[229, 260]	0.58
	10^{-7}	5	-3	[-11, 4]	n.a.	129	[119, 139]	0.5	192	[178, 207]	0.32	240	[223, 257]	0.33
	10^{-6}	5	1	[-13, 14]	n.a.	122	[112, 132]	0.13	183	[169, 197]	0.035	229	[213, 245]	0.032
	10^{-5}	5	2	[-14, 17]	n.a.	120	[110, 129]	0.063	181	[168, 194]	0.026	227	[213, 242]	0.025

BL, baseline

RM, ANOVA repeated measures & LSD Post-Hoc testing

FA, Friedman ANOVA & Wilcoxon pairwise testing

1, p-values are computed for RM/FA and individual groups compared to baseline

n.a. indicates missing data/lack of post-hoc testing
